# Inter-hospital transport of critically ill patients to manage the intensive care unit surge during the COVID-19 pandemic in France

**DOI:** 10.1186/s13613-021-00841-5

**Published:** 2021-03-31

**Authors:** Benoit Painvin, Hélène Messet, Maeva Rodriguez, Thomas Lebouvier, Delphine Chatellier, Louis Soulat, Stephane Ehrmann, Arnaud W. Thille, Arnaud Gacouin, Jean-Marc Tadie

**Affiliations:** 1 Service de Réanimation Médicale et des Maladies Infectieuses, Centre Hospitalier Universitaire de Rennes, Hôpital Pontchaillou, 2 rue Henri le Guilloux, 35033 Rennes Cedex 9, France; 2 Service de Médecine Intensive et Réanimation, Centre Hospitalier Régional Universitaire de Tours, Hôpital Bretonneau, 2, boulevard Tonnellé, 27044 Tours cedex 9, France; 3 Service de Médecine Intensive et Réanimation, Centre Hospitalier Universitaire de Poitiers, 2 rue de la Milétrie, CS 90577, 86000 Poitiers, France; 4 Service de Réanimation Chirurgicale, Centre Hospitalier Universitaire de Rennes, Hôpital Pontchaillou, 2 rue Henri le Guilloux, 35033 Rennes Cedex 9, France; 5 Service Samu-Smur-Urgences médico-chirurgicales adultes, Centre Hospitalier Universitaire de Rennes, Hôpital Pontchaillou, 2 rue Henri le Guilloux, 35033 Rennes Cedex 9, France; 6 Faculté de Médecine, Université de Rennes 1, Unité INSERM CIC 1414, IFR 140, Rennes, France; 7 Service de Médecine Intensive et Réanimation, CIC INSERM 1415, CRICS-Triggersep Research Network, Centre Hospitalier Régional Universitaire de Tours, Hôpital Bretonneau, 2, boulevard Tonnellé, 27044 Tours cedex 9, France; 8 Centre d’étude des Pathologies Respiratoires, INSERM U1100, Université de Tours, Tours, France

**Keywords:** COVID-19, Intensive care unit, Hospital transport, Mechanical ventilation, ARDS, Mortality

## Abstract

**Background:**

The COVID-19 pandemic led authorities to evacuate via various travel modalities critically ill ventilated patients into less crowded units. However, it is not known if interhospital transport impacts COVID-19 patient’s mortality in intensive care units (ICUs). A cohort from three French University Hospitals was analysed in ICUs between 15th of March and the 15th of April 2020. Patients admitted to ICU with positive COVID-19 test and mechanically ventilated were recruited.

**Results:**

Among the 133 patients included in the study, 95 (71%) were male patients and median age was 63 years old (interquartile range: 54–71). Overall ICU mortality was 11%. Mode of transport included train (48 patients), ambulance (6 patients), and plane plus helicopter (14 patients). During their ICU stay, 7 (10%) transferred patients and 8 (12%) non-transferred patients died (*p* = 0.71). Median SAPS II score at admission was 33 (interquartile range: 25–46) for the transferred group and 35 (27–42) for non-transferred patients (*p* = 0.53). SOFA score at admission was 4 (3–6) for the transferred group versus 3 (2–5) for the non-transferred group (*p* = 0.25). In the transferred group, median PaO_2_/FiO_2_ ratio (*P*/*F*) value in the 24 h before departure was 197 mmHg (160–250) and remained 166 mmHg (125–222) in the first 24 h post arrival (*p* = 0.13). During the evacuation 46 (68%) and 21 (31%) of the patients, respectively, benefited from neuromuscular blocking agents and from vasopressors. Transferred and non-transferred patients had similar rate of nosocomial infections, 37/68 (54%) versus 34/65 (52%), respectively (*p* = 0.80). Median length of mechanical ventilation was significantly increased in the transferred group compared to the non-transferred group, 18 days (11–24) and 14 days (8–20), respectively (*p* = 0.007). Finally, ICU and hospital length of stay did not differ between groups.

**Conclusions:**

In France, inter-hospital evacuation of COVID-19 ventilated ICU patients did not appear to increase mortality and therefore could be proposed to manage ICU surges in the future.

## Background

In early 2020, Coronavirus infectious disease (COVID-19) emerged as a worldwide pandemic. France, as many countries, faced a large number of hospital admissions for patients with COVID-19 [1–3]. A French cohort of 4244 patients admitted to the intensive care unit (ICU) for COVID-19 reported a lengthy duration of mechanical ventilation (MV) of 13 days and a high overall 90-day mortality of 31% [4]. These clinical observations and the surge of severe COVID-19 cases led countries to rearrange hospital work forces, to treat critically ill patients outside of the ICU and to build new health facilities [5–9]. French authorities decided to transfer critically ill COVID-19 patients from overcrowded critical care wards, notably East of France and Paris area, to regions with better ICU bed capacities such as West of France which was partially preserved during the first epidemic surge. High-speed trains, planes, helicopters, and ambulances were commissioned to transport mechanically ventilated patients. However, transport of critically ill patients could increase MV duration, length of ICU stay and mortality [10–12]. Considering the importance of evaluating such strategy, we performed a retrospective study to assess outcomes of critically ill COVID-19 patients transferred from an overcrowded ICU to the ICUs in the West of France.

## Patients and methods

Data were retrospectively analysed from medical records in 3 teaching hospitals in the West of France (Poitiers, Rennes, and Tours). All patients above 18 years old admitted to these 3 ICUs from the 15th of March until the 15th of April and requiring invasive mechanical ventilation for COVID-19 confirmed by reverse transcriptase-polymerase chain reaction test were included. The study has been approved by the local ethics committee which waived the need for informed consent according to the French legislation (Institutional Review Board of the Rennes University Hospital, No. 20.65).

The main objective was to compare the ICU mortality between patients who underwent inter-hospital transfer during their ICU stay and patients who did not. Secondary objectives were the comparisons in ICU-acquired infections, length of mechanical ventilation, length of sedation and use of neuromuscular blocking agents, length of vasopressor use, ICU and hospital length of stay between the two groups.

### Data collection

We collected demographic data, comorbidities, and severity score such as the Simplified Acute Physiologic Score II (SAPS II) [13] and the Sequential Organ Failure Assessment (SOFA) score [14]. In transferred patients, severity scores were also measured during the first 24 h following the transfer. Acute respiratory distress syndrome (ARDS) and its severity was defined according to the Berlin definition [15]. We documented the PaO_2_/FIO_2_ (*P*/*F*) ratio, duration of MV, the occurrence of ARDS and its severity, and the necessity for prone positioning. We recorded throughout patients’ ICU stay the occurrence of acute renal failure according to the acute kidney injury network (AKIN) [16], the need for renal replacement therapy, and the length under sedatives, opioids and neuromuscular blocking agents. Implantation of veno-venous extra-corporeal membrane oxygenation (ECMO) and its duration, acquired infections, thrombotic events, length of ICU and hospital stay, and ICU and hospital mortality were recorded.

### Statistical analysis

Continuous variables were compared using the Student’s *t*-test or the Mann–Whitney *U* test according to their distribution and categorical variables were compared using the Chi^2^ test or the Fisher’s exact test as appropriate. Data before and after transfer were compared with *t*-test on paired variables. We adjusted mortality comparison between the two groups with occurrence of AKI, proning position, and duration of mechanical ventilation. A two-tailed *P* value of less than 0.05 was considered to indicate statistical significance. Analyses were performed with Stat View® 5. software.

## Results

Over a 1-month period, 133 patients with COVID-19 under invasive mechanical ventilation were studied, including 47 (35%) patients in Rennes, 43 (32%) patients in Tours and 43 (32%) patients in Poitiers. Among them, 68 (51%) were transferred patients, including 29 patients transferred to Rennes, 9 to Tours and 30 transferred to Poitiers. Rennes and Poitiers are situated at 350 km from Paris and Tours at 260 km from Paris. Transferred patients’ median ICU length of stay prior to transportation was 7.5 days (interquartile 4–11 days). Forty-eight, 14, and 6 patients were transported by train, air, and ambulances, respectively. Nurse/patient and doctor/patient ratio were 1/1 and ½, respectively, for both train and plane transport. Helicopter transfer had ratios of one nurse and one doctor per patient. Twenty-four hours before departure, all transferred patients were sedated. Thirty-six patients (53%) were under neuromuscular blocker infusion and 16 patients (23.5%) under vasopressor infusion.

The baseline characteristics of the patients are reported in Table 1. Comorbidities were similar in both groups.

**Table 1:** Baseline values and outcomes for transferred and non-transferred patients

	Transferred *n* = 68	Non-transferred *n* = 65	*P* value
Age, median (IQR), year	62 (55–70)	64 (54–71)	0.77
Male sex, no. (%)	47 (69)	48 (74)	0.54
BMI, median (IQR), kg/m^2^	29 (25–32)	29 (27–33)	0.27
SAPS II score at admission^a^, median (IQR)	33 (25–46)	35 (27–42)	0.53
SOFA score at admission^a^, median (IQR)	4 (3–6)	3 (2–5)	0.25
Onset of symptoms before ICU admission, median (IQR), days	9 (7–12)	10 (8–12)	0.34
Alcohol chronic intoxication, no. (%)	3 (3)	7 (11)	0.09
Cardiovascular comorbidities, no. (%)	36 (53)	34 (52)	0.94
Respiratory comorbidities, no. (%)	13 (19)	15 (23)	0.56
Renal comorbidities, no. (%)	5 (7)	7 (11)	0.56
Metabolic comorbidities, no. (%)	27 (38)	24 (37)	0.74
Malignant comorbidities, no. (%)	4 (6)	7 (11)	0.36
Chronic betablockers medication, no. (%)	15 (22)	9 (14)	0.22
Chronic ACE inhibitors medication, no. (%)	23 (35)	23 (34)	0.85
Chronic corticosteroids medication, no. (%)	3 (4)	3 (5)	0.99
Patients’ outcome and support at admission and over stay in Poitiers, Rennes, and Tours ICUs			
Proning, no. (%)	24 (35)	42 (65)	0.001
Number of proning session(s), median (IQR)	2.5 (1.5–4)	3 (1–5)	0.43
Implantation of veno-venous ECMO, no. (%)	5 (7)	7 (11)	0.49
Length of veno-venous ECLS, median (IQR), days	18 (6–38)	9 (7–12)	0.52
Vasopressors use, no. (%)	44 (65)	45 (69)	0.58
Occurrence of AKI, no. (%)	18 (26)	29 (45)	0.03
Need for RRT, no. (%)	8 (12)	9 (14)	0.79
Occurrence of bacteremia, no. (%)	10 (15)	5 (8)	0.31
Occurrence of thrombotic event, no. (%)	25 (37)	20 (31)	0.54
Primary and secondary outcomes			
ICU mortality, no. (%)	7 (10)	8 (12)	0.71
Hospital mortality, no. (%)	8 (12)	8 (12)	0.92
ICU-acquired infections, no. (%)	37 (54)	34 (52)	0.82
Length of mechanical ventilation, median (IQR), days	18 (11–24)	14 (8–20)	0.007
Length of intra-venous sedation, median (IQR), days	10 (6–17)	11 (6–18)	0.57
Duration of neuromuscular blocking agents, median (IQR), days	2 (1–5)	5 (3–9)	0.008
Length of vasopressor use, median (IQR), days	2.5 (2–5)	3 (2–5)	0.77
ICU length of stay, median (IQR), days	22 (16–32)	19 (11–28)	0.07
Hospital length of stay, median (IQR), days	36 (24–48)	30 (19–45)	0.11

*AKI* acute kidney injury, *BMI* body mass index, *ECMO* extra-corporeal membrane oxygenation, *ICU* intensive care unit, *IQR* interquartile range, *RRT* renal replacement therapy, *SAPS* simplified acute physiology score, *SOFA* sequential organ failure assessment

^a^Scores calculated at initial admission in Rennes, Poitiers, and Tours for the non-transferred group and in Paris and East of France for the transferred group

During the evacuation, 68 patients were transferred. Among them, 46 (70.7%) were under neuromuscular blockers infusion. Among these 46 patients, 10 (21.7%) were put under neuromuscular blockers specifically for transportation. Regarding vasopressor initiation or increase during transport, 20 (30.7%) were under vasopressors infusion during transportation. Among these 20 patients, 4 (20%) had initiation of vasopressors or an increase in vasopressor dose during transport.

PaO_2_/FiO_2_ ratio, measured within the 24 h before departure and within 24 h after transfer, did not significantly differ [197 mmHg (interquartile range, IQR 160–250) vs. 166 mmHg (IQR 125–222), respectively, *p* = 0.13]. Similarly, SOFA score 24 h prior transportation did not significantly vary compared to post-transfer SOFA score [6 (IQR 4–8) versus 7 (IQR 4–9), respectively, *p* = 0.07]. One patient was transported by ambulance with a veno-venous ECMO due to pulmonary embolism and died 24 h after admission. One patient transferred by train, needed an implantation of a veno-venous ECMO less than 24 h after its arrival in ICU. No patient died during interhospital evacuations and no complication occurred (i.e. catheter accidental removal, extubation).

ICU mortality was 11% for the whole population and did not differ significantly when compared between transferred and not transferred patients, 7 (10%) and 8 (12%) (*p* = 0.71), respectively (Table 1). After adjustment for AKI, number of proning session and duration of MV, the difference of mortality between the two groups was non-significant with an adjusted odds ratio of 1.2, (95% confidence interval 0.35–4.09) (*p* = 0.76). With regard to secondary outcomes, median length of MV was significantly longer in the transferred group compared to the non-transferred group, 18 days (11–24) and 14 days (8–20) (*p* = 0.007), respectively. We also found that ICU length of stay was comparable between the two populations: 22 days (16–32) in the transferred group and 19 days (11–28) in the non-transferred group (*p* = 0.07). Other secondary outcomes are detailed in Table 1.

## Discussion

In our study, we showed that COVID-19 ICU patients who were initially hospitalized for respiratory failure and who required interhospital transport did not suffer higher mortality rates when compared to COVID-19 ICU patients admitted locally. However, the length of mechanical ventilation was increased in the transferred group.

Worldwide, the Sars-CoV-2 pandemic led ICU bed capacities to be exceeded. Urgent solutions such as treating patients with respiratory distress in conventional units using noninvasive respiratory support appeared feasible yet with a higher risk of staff contamination [9]. Other temporary measures included telecritical care service to support newly formed ICU personnel [17]. Consequently, health authorities set up two main long-term strategies: relocate work forces and construct new medical capacities and/or transfer critically ill patients to less busy areas [6, 7, 18]. The occurrence of a second and possible third wave of COVID-19 necessitated a rapid evaluation of these strategies.

Patients’ transfers between ICUs are not risk-free and can increase ICU and hospital length of stay [10, 19, 20]. Similarly, intrahospital transfer of critically ill patients is associated with pneumothorax, atelectasis, ventilator-associated pneumonia, dysglycemia, and a longer ICU length of stay in a French cohort of 6000 patients [11]. Conversely, mortality does not seem to be impacted by intra- and inter-hospital transfers [11, 19, 20]. In France, authorities opted for medical transfer of ICU patients, predominantly via train, due to France large rail network: indeed, it operates the second-largest European railway network, with a total of 29,900 km of railway of which 2600 km are high-speed lines [21]. Moreover, this transfer strategy was also feasible because of the network of 32 tertiary centres covering French territory, all of them being located and served by an airport and train station [22].

Regarding specific medical transport of COVID-19 ventilated patients, the literature is scant and most of it describes the process and the means of transporting COVID-19 patients rather than exploring their clinical and biological features [23, 24]. One study by Boutonnet et al. described the procedure of evacuating 36 COVID-19 patients via French Air Force military planes. They described that two-thirds of their patients received catecholamine infusion, yet none encountered life-threatening event during flight [25].

In our study the ICU mortality rate was 11%, far lower than from recent studies with large populations where the mortality rate oscillated between 30 and 50% [1, 3, 4]. Several factors could explain this discrepancy. Firstly, Rennes, Poitiers, and Tours Intensive Care Units (and by extension hospitals), were far from being overwhelmed during the study period compared to other regions in France. In fact, transferred patients accounted for nearly half of the COVID-19 patients’ admission during the study period. It enabled doctors and staff to promptly admit patients, to take time to make critical decisions such as the decision to intubate, initiate treatments, or extubate. Secondly, transferred patients were admitted in real ground concrete medical and surgical ICUs, with highly trained staff, nurses, and physicians. Thirdly, selection bias is likely to play a role in the low ICU mortality rate as most transferred patients were clinically stable before departure. Finally, because of a delay in surge of COVID-19 cases in Rennes, Poitiers, and Tours ICU, clinicians benefited from other centres experience regarding COVID-19 management, complications, and care.

Along these lines, in a recent study by Taccone et al., ICU overflow and having a high proportion of created ICU beds were independently associated with in-hospital mortality [26]. One solution to face the next pandemic may be to increase the average number of ICU beds per inhabitant ensuring homogenous distributions across territories [27].

In addition to our main result, we showed that in the transferred group, PaO_2_/FiO_2_ ratio and SOFA score were similar immediately before and after transport. These findings highlight that in the COVID-19 patient population, treated for predominant respiratory illness with underlying ARDS, medical transfer did not appear to worsen their general state and specifically their respiratory function.

Furthermore, acquired infection, length of ICU and hospital stay were similar in both groups. However, length of mechanical ventilation was increased in the transferred group. This could be explained by a stop in the invasive mechanical ventilation weaning process because of the upcoming interhospital transfer and the necessity for sedation during transfer. These results may lead to the hypothesis that the need to sedate patients during transport will therefore increase the length of stay without worsening the outcome.

Following the analysis and reflections surrounding our study results, we can state that transport of COVID-19 ICU patients is safe and constitutes a possible management to face urgent and locally overwhelmed hospitals and ICU capacities. Transferring patients into non-overwhelmed areas or transferring healthcare workers into overwhelmed areas are both valuable strategies to face the pandemic. Unfortunately, efficacy, cost, and results of these two strategies have not been evaluated yet.

Our study has several limitations, including data being collected retrospectively, small number of patients and the transfer of patients’ medical file with the risk of loss of information. Furthermore, selection bias may have been introduced in choosing which patients were transferred. It is noteworthy that transferred patients have probably been selected and that the ideal control group should have been non-transferred patients from the same areas. However, at their initial admission, severity scores were identical between both groups of patients. Next, due to missing information, calculation of neuromuscular blockers duration did not include transferred patients’ pre-transportation data, hence comparison of this value between the two groups must be undertaken cautiously.

In conclusion, we found that interhospital medical transfer of mechanically ventilated COVID-19 critically ill patients did not result in a higher mortality, but did increase length of mechanical ventilation. This could be proposed as a safe strategy to manage the surge of ICU needs in the future.

## Data Availability

The datasets used and/or analysed during the current study are available from the corresponding author on reasonable request.

## Abbreviations

ARDS: Acute respiratory distress syndrome
COVID-19: Coronavirus infectious disease
ICU: Intensive care unit
IQR: Interquartile range
MV: Mechanical ventilation
SOFA: Sequential Organ Failure Assessment
SAPS II: Simplified Acute Physiologic Score II
ECMO: Extra-corporeal membrane oxygenation
